# p53, A Victim of the Prion Fashion

**DOI:** 10.3390/cancers13020269

**Published:** 2021-01-13

**Authors:** Olivier Billant, Gaëlle Friocourt, Pierre Roux, Cécile Voisset

**Affiliations:** 1 CRBM, CNRS, UMR5234, 34293 Montpellier, France; pierre.roux@crbm.cnrs.fr; 2 Inserm, Université de Bretagne Occidentale, EFS, UMR 1078, GGB, F-29200 Brest, France; Gaelle.friocourt@univ-brest.fr

**Keywords:** p53, isoform, prion, aggregation, amyloid, PMD

## Abstract

**Simple Summary:**

The tumor suppressor gene *TP53* is found mutated in around half of human cancers. The accumulation of the mutant p53 protein in the form of aggregates in cancer cells have led to the emergence of the “prion p53” hypothesis which states that mutant p53 is able to drive the wild-type form of the protein into an alternate conformation, thereby contributing to tumor progression. This report challenges the “prion p53” hypothesis by reviewing evidence of p53 behavior in light of our current knowledge regarding amyloid proteins, prionoids and prions.

**Abstract:**

Identified in the late 1970s as an oncogene, a driving force leading to tumor development, p53 turned out to be a key tumor suppressor gene. Now p53 is considered a master gene regulating the transcription of over 3000 target genes and controlling a remarkable number of cellular functions. The elevated prevalence of p53 mutations in human cancers has led to a recurring questioning about the roles of mutant p53 proteins and their functional consequences. Both mutants and isoforms of p53 have been attributed dominant-negative and gain of function properties among which is the ability to form amyloid aggregates and behave in a prion-like manner. This report challenges the ongoing “prion p53” hypothesis by reviewing evidence of p53 behavior in light of our current knowledge regarding amyloid proteins, prionoids and prions.

## 1. Introduction

**Figure 1:**
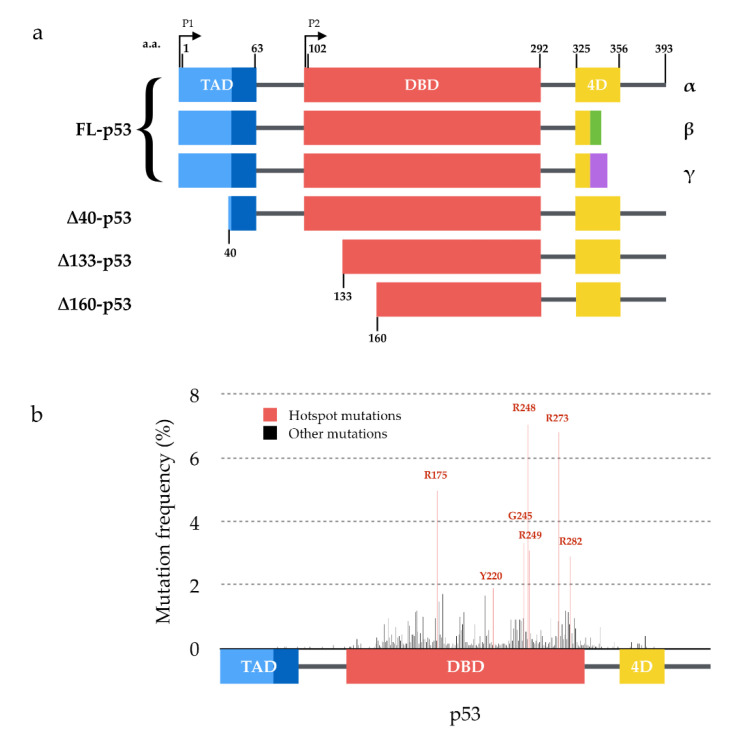
Structure of p53 isoforms and distribution of mutations within p53. (**a**) p53 isoforms are generated from the use of two alternative promoters (P1 and P2), secondary initiation codons (∆40, ∆133 or ∆160) and/or alternate splicing sites (α, β or γ C-termini) leading to 12 combinations described to date. (**b**) Distribution and frequency of p53 mutations described in human tumors (data from the International Agency for Research on Cancer). Hotspot mutations are shown in red and are all located within the DNA binding domain of p53.

p53 is a 393-amino-acids-long transcription factor composed of a globular DNA binding domain flanked by a transcription activation domain in N-terminal and a tetramerization domain in the C-terminal part of the protein (Figure 1a) [1] and is active in a homo-tetrameric state [2]. p53’s best-described functions mainly revolve around genome maintenance. p53 is usually kept at a low basal protein level by the E3-ubiquitin ligase MDM2 (murine double minute 2) and its paralog MDM4 [3]. Following DNA damages or other cellular stresses, p53 level rises as the protein is phosphorylated and stabilized, which in turn facilitates DNA repair by pausing the cell cycle (via p21) or guides damaged cells toward apoptosis (via BAX) [4,5,6].

However, the progressive accumulation of data regarding p53 target genes has highlighted the fact that the p53 response is remarkably flexible and that it regulates different sets of genes depending on the cell type, stress conditions and microenvironment signaling. p53 indeed participates in autophagy, senescence, metabolism, proliferation, differentiation, immune response and inflammation [7,8,9,10,11,12]. The complexity of the characterization of p53 functions is also increased by the fact that the *TP53* gene encodes at least 12 isoforms (Figure 1a), whose roles remain to be fully unraveled [13,14,15]. In addition, the identification of two p53 paralogues, p63 [16] and p73 [17,18], which also encode multiple isoforms [19], adds further complexity to an already dense network. Both p63 and p73 display a modular structure similar to that of p53 with which they also share a high degree of similarity at the protein level [20,21]. This leads to the regulation by these three genes of a common set of target genes involved in apoptosis and cell cycle arrest, and highlights p63 and p73 as candidate tumor suppressor genes [22].

Given the broad spectrum of p53’s functions, its inactivation obviously places a heavy burden on the cell. Mutations of p53 are indeed found in about half of human tumors on average [23,24]. The vast majority of these mutations are amino acid substitutions, primarily located in the DNA binding domain (Figure 1b). The functional consequences of p53 mutations are manifold. In particular, mutant p53 proteins have been shown to be less efficiently ubiquitinated [25] and can thus be stabilized by evading proteasomal degradation through the MDM2 degradation loop, thereby increasing their cellular levels [26]. Several mutations are considered to confer gain-of-function properties to p53 (e.g., increased protein half-life, activation of additional downstream pathways) and have been associated with poorer prognosis for the patients [26,27,28,29,30]. When the mutations are heterozygous, p53 mutant proteins have notably been shown to interfere by a dominant-negative effect with the transcriptional activity of the wild-type p53 proteins encoded by the remaining wild-type allele [14,31,32,33,34]. Among the mechanisms proposed to explain the dominant-negative effect of mutant p53 over wild-type p53, the “prion-like” hypothesis has been steadily gaining momentum during the past decade. Twenty-five years after this hypothesis was first introduced, there is now enough data to review this theory. We have scrutinized the current research dedicated to deciphering the ability of p53 dominant-negative mutants to actually behave in a prion-like manner. To begin with, we will define the terms amyloid, prion and prion-like in order to clearly lay the foundations of our line of argument.

## 2. Amyloid, Prion and Prionoids

### 2.1. Members Only, the Defining Traits of Amyloid Proteins

One of the defining traits of prion and prion-like proteins is their amyloid structure. The term amyloid was coined in 1838 by botanist Matthias Schleiden (from the Latin *amylum* (starch)) to describe an amylaceous constituent of plants. In 1854, Rudolph Virchow first used the term amyloid because of the peculiar reaction of iodine with the *corpora amylacea* of the nervous system, which he first mistakenly took for starch [35].

In vivo amyloids share three main hallmarks [36]. Firstly, they display a fibrillary morphology. As the amyloid architecture is a consequence of the physicochemical properties of a polypeptide chain, a given peptide sequence can be incorporated in fibrils in multiple ways and give rise to various fibril morphologies. Secondly, amyloids are formed by proteins enriched in β-sheet secondary structure as a result of conformation change. These proteins then adopt a cross-β structure in which β-strands are oriented perpendicularly to the fibril axis and are assembled into β-sheets that run the length of the fibrils with a parallel or antiparallel in-register arrangement. Only a fraction of the polypeptide chain is incorporated in the cross-β core of the fibrils. Thirdly, amyloids specifically bind dyes, such as thioflavin (ThT or ThS) or Congo Red [36,37]. Any given protein aggregate needs to display these three features to be classified as amyloid in vivo. Remarkably, said fibrils are formed by polypeptide chains which have no similarity in sequence, structure or function whatsoever, nevertheless sharing morphological and structural properties. The term amyloid has thus evolved into a polysemous term as it refers to protein-based structures associated with protein misfolding diseases (PMDs), as well as to a wide panel of essential functions performed by proteins called functional amyloids [38].

So far, among the large panel of amyloid-forming proteins, 36 proteins and peptides (e.g., PrP, α-syn, IAPP) have been formally proven to form pathological amyloid in vivo [36]. Among the pathological amyloids, prion and prionoid have the property of being able to self-replicate; but the prion protein PrP presents the seemingly unique property of being inter-individually transmittable.

### 2.2. PrP, Still the Only One in the Prion Category

The term prion refers to the proteinaceous agent causing prion diseases, now known to be PrP^Sc^, the pathological isoform of the cellular prion protein PrP^C^, which is encoded by the *PRNP* gene [39,40]. Prions are thought to multiply by a nucleation and fragmentation process during which PrP^Sc^ oligomers grow in size through incorporation of endogenous PrP^C^ [41]. Large PrP^Sc^ aggregates may then be taken apart into smaller fragments, called propagons, able to singlehandedly initiate a new nucleation–fragmentation cycle [42,43]. PrP^C^ protein can adopt various PrP^Sc^ self-propagating conformations, each being able to propagate its biochemical signature to naive PrP^C^ proteins. The existence of these structurally distinct assemblies is referred to as prion strains and leads to different molecular, histopathological and clinical phenotypes (reviewed in [44]).

Prion diseases are also called transmissible spongiform encephalopathies (TSE) as they have all the characteristics of infectious diseases, such as transmissibility, species barriers, as well as the existence of strains. Prion diseases are found in several mammalian species, among which cattle, where the mad cow disease pandemic originated in the late 1990s [45,46], and cervids whose wild population is increasingly affected by the chronic wasting disease (CWD) in North America wooded areas and more recently in Europe [47]. Prion diseases also affect humans with an incidence of 1–2 cases per million. Human prion diseases can be either genetic, sporadic or acquired. Genetic prion diseases like genetic Creutzfeldt–Jakob disease (gCJD), fatal familial insomnia (FFI) and Gerstmann–Straussler–Scheinker syndrome (GSS) account for 5% of cases and are all caused by mutations in the *PRNP* gene (for review see [48]). Sporadic prion diseases like sCJD and sporadic FFI represent 85% of cases and have yet unknown etiology. Acquired prion diseases are due to the transmission of prions within the human species (through medical procedure, growth hormone supplementation, cannibalism) or from animal to human (for example by the consumption of products derived from cattle suffering from “mad cow” disease, leading to vCJD). All these prion diseases are characterized by the accumulation of PrP^Sc^ amyloid aggregates in the central nervous system.

### 2.3. Prionoids, the New Challengers

An increasing number of neurodegenerative disorders, including Alzheimer disease (AD), Parkinson disease (PD), amyotrophic lateral sclerosis (ALS), Huntington’s disease (HD), and also metabolic diseases like type 2 diabetes (T2D) and AA amyloidosis A, have been linked to protein misfolding and aggregation [41,49,50,51,52]. In these PMDs, also often termed “prion-like diseases,” specific proteins are detected under the form of amyloid fibers in patients’ tissues, just as PrP^Sc^ (e.g., Aβ and tau in AD, α-Synucelin in PD, TDP-43 in ALS, HTT in HD, IAPP in T2D). The clinical consequences of their misfolding vary between these diseases and can occur in different organs, among which the central nervous system, liver, spleen, pancreas or the peripheral nervous system. Yet, all PMD-associated proteins are found in extracellular and/or intracellular amyloid aggregate deposits and share critical features with prion proteins (for review, [50,53,54,55]). Firstly, alternatively folded or partially unfolded isoforms of a disease-associated protein interact with each other to form cross-β spines [56] that assemble into propagons able to self-propagate [42]. Propagons grow into protofilaments that interact with each other to form higher-order fibrillary aggregates. Fibrils can also be fragmented, which frees up additional seeds that possess templating (and therefore self-perpetuating) activity of their own [41]. Secondly, PMD-associated proteins are capable of cell-to-cell propagation in vitro, and aggregates have also been shown to seed and spread upon inoculation and initiate disease in vivo [55,57]. Thirdly, PMD-associated proteins also display strains and species barriers [55,58,59,60,61,62].

Because PMD-associated amyloids resemble prions in their self-templating propagation, cell-to-cell transmissibility and existence of species barrier and strains, PMD-associated amyloids are often referred to as prion or prion-like proteins. However, inter-individual transmissibility of PMD-associated proteins, which is a defining trait of prions, has not been definitively proven yet. The term “prionoid” thus emerged as a way to classify non-infectious self-propagating amyloid aggregates capable of cell-to-cell propagation within individuals [41,49,50].

A given prionoid might however change classification if its infectivity happens to be conclusively demonstrated. Notably, some evidence of the experimental transmission of protein aggregates at cellular and organism levels are emerging regarding Aβ [63,64,65], α-Synuclein [66], tau [67], and amyloid A seeds [68,69,70,71]. Further studies exploring the natural donor/host transmission are eagerly awaited as they should assess whether or not certain prionoids are also transmissible or if prions are still the only protein agents capable of actually transmitting diseases in a natural environment.

### 2.4. Functional Amyloids, Cellular Multitools

The term amyloid is essentially associated with neurodegenerative diseases and systemic amyloidosis, and is thus perceived as negative and deleterious for the cell. However, knowledge about functional amyloids is emerging and certain amyloid assemblies have now been described as performing a variety of essential life processes in many organisms [38]. In mammals, functional amyloids are, for instance, involved in the chemical storage of peptide hormones [72] and of the blood clotting proteins fibrin [73], the scaffolding avoiding melanin toxicity (Pmel17, [74]), the long-term memory (CPEB3, [75]), mouse fertility (CRES genes, Cornwall 2019 Andrology) and the necroptosis signaling in virus-infected cells (RIP1/RIP3, [76]). But essential functions are also driven by amyloids or super-assemblies in other organisms, like biofilm formation in *Escherichia coli* (Curli) and *Bacillus subtilis* (TasA), plasmid replication control in Bacteria (RepA, [77]), long-term memory in *Aplysia* (CPEB, [78]) and *Drosophila* (Orb2), memory of past unsuccessful mating encounters leading to aging in *Saccharomyces cerevisiae* Whi3, [79,80]), protection of germline components in dormant oocyte in *Xenopus laevis* (Xvelo, [81,82]), *Drosophila* (Oskar), Zebrafish (Bucky ball) and *Caenorhabditis elegans* (P granules) or immune response in *Drosophila* (Imd, [83]). HET-s, which is involved in heterokaryon incompatibility in *Podospora anserina,* has a special place in the amyloid world as it is a functional amyloid with prion characteristics [84]. It is now accepted that protein aggregation actually plays a native role in cellular functions. The high stability of the amyloid structures indeed provides structural organizing scaffolds which can be easily and quickly mobilized in response to environmental and physiological conditions.

Despite their structural similarities, the kinetics of aggregation of pathological and functional amyloids diverge, the formation of pathological amyloids being much slower than the fibrillation of functional ones. Moreover, a specificity of functional amyloids is that their formation and disappearance are tightly controlled as they can be disintegrated and release functional monomers on demand. Amyloid formation is thus not necessarily an irreversible process as it is thought to be for prions and prionoids. It is important to note that the pathological entities in prions and prionoid-based diseases are most probably the small oligomers and not the amyloid fibers *per se* [85,86]. Large fibrils can have a buffering effect by sequestering toxic oligomers within more inert structures [87], which makes them comparable to certain functional amyloids, the quick aggregation kinetics of which may spare cells from oligomer toxicity.

Prion, prionoid and amyloid entities are now more clearly defined in the literature and although putting p53 in a box is not the point of this review, it is high time to reassess its actual prion-like properties.

## 3. Prion p53, the Origin Story

The first mention of a “prion p53” actually dates back to 1995 [88]. As a basis to explain the dominant-negative effect of mutant p53 over the wild-type protein, Jo Milner and Elizabeth Medcalf evaluated the in vitro effect of several mutants of p53 (p. R151S, p. R247I, p. R273P, p. R273L) on wild-type p53. They showed that these mutants drive a wild-type p53 bearing a “wild-type” conformation [p53^WT^] toward a mutant conformation [p53^MUT^] when co-translated, thereby discriminating two in vitro allosteric variants of wild-type p53 [89].

**Figure 2:**
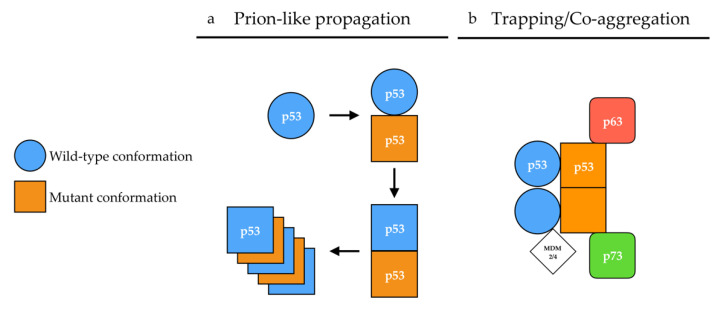
Proposed mechanisms of p53 aggregation. (**a**) According to the prion-like hypothesis, mutant p53 (orange) in a [p53^MUT^] conformation (square) induces a conformational shift of wild-type p53 (blue) from a [p53^WT^] (circle) conformation toward a [p53^MUT^] conformation in a prion-like manner. In a [p53^MUT^] conformation, wild-type and mutant p53 form amyloid fibers. (**b**) According to the co-aggregation/trapping hypothesis, mutant p53 hetero-tetramerizes with wild-type p53. The unfolded core of mutant p53 allows new interactions with p63/p73 isoforms and MDM2/4 in a trapping/co-aggregation mechanism.

The conformational flexibility of p53 has also provided grounds to the prion p53 hypothesis. Changes in p53 conformation have been monitored by their reactivity to conformation-specific antibodies [90]. For example, pAb1620 antibody recognizes the [p53^WT^] conformation while pAb240 [91] recognizes the [p53^MUT^] conformation. From there on, p53 mutants were classified as having wild-type [p53^WT^] or mutant [p53^MUT^] conformations based on their reactivity with either pAb1620 or pAb240 antibody. Mutants harboring a [p53^WT^] conformation were, however, not systematically transcriptionally active. These observations later gave rise to the subdivision of p53 mutants into two categories: (i) “DNA contact” mutants (R273H, R248Q, R248W), which have a decreased ability to bind DNA, and (ii) “conformation or structural” mutants (R175H, G245S, R249S, R282H), in which mutations induce a local to global destabilization of the protein structure [88,92] that exposes specific motives at the surface of the protein, thereby allowing reactivity with the pAb240 antibody. However, the relevance of these two classes of mutants is being challenged [93,94]. Some mutations such as p.V135A lead to a [p53^WT^] conformation when translated at 30 °C, but the protein undergoes a conversion to a [p53^MUT^] conformation within two minutes when the temperature shifts from 30 °C to 37 °C [95], also illustrating p53 conformational flexibility. Hence, the apparent ability of some p53 mutants to induce a conformational shift of a wild-type p53 protein bearing a [p53^WT^] conformation towards a [p53^MUT^] conformation, together with the existence of alternative conformations of p53 gave ground to the “prion p53” hypothesis [88,96], at a time when the prion protein PrP^Sc^ was in the limelight (Figure 2a).

### 3.1. p53 Aggregation in Tissue Samples and Tumor Cell Lines

p53 mutants have early on been described as forming aggregates in tissue samples and tumor cell lines. p53 has indeed been shown to form protein aggregates that abnormally accumulate in the nucleus and sometimes in the cytoplasm of cells from several types of tumor samples [97,98,99,100,101,102]. The aggregative features of p53 have been monitored for a time by immunohistochemistry (anti-p53 antibodies DO-1 and others) as a surrogate for the identification of tumors carrying p53 mutation [98,103,104,105], which is associated with worsened cancer prognosis (reviewed in [106]). However, immunohistochemistry often fails to deliver consistent results and is thus not the expected gold standard [28,104].

The amyloid nature of p53 aggregates has been mostly explored by co-localization experiments relying on anti-p53 antibodies, anti-fibrils antibodies (OC, A11 [107,108,109]) or amyloid dyes (thioflavin T, thioflavin S or Congo red). However, major variations are observed in the quantity and features of p53 aggregation, depending on the cell type, the mutation of p53 or the method used to quantify said aggregation [106,110,111,112,113,114], thereby rendering arduous any extensive comparison. In addition, the nature of the proteins forming the A11/OC-positive or amyloid dye-positive protein aggregates was not determined, thus the sometimes-partial co-localization of p53 with these amyloid aggregates precludes, for now, p53 from being classified as a genuine amyloid by the International Society of Amyloidosis nomenclature committee [36,115].

Studies concerning p53 aggregation have been limited to the canonical (full-length FL-p53α) isoform of p53 and its mutant forms. However, recent reports focused on the potential aggregation of p53 isoforms ∆40p53 [116] and ∆133p53 [117,118] have opened new perspective to the field. The tissue-specific expression of p53 isoforms [15] could indeed partially account for the diversity of p53 aggregation phenotypes observed.

### 3.2. p53 Aggregation: In Vitro Dynamics

Despite the fact that only 36 proteins have been shown to form pathogenic amyloids in vivo, it is important to note that in vitro amyloid formation is observed for many more protein sequences. It is actually a property shared by many, if not all, natural polypeptide chains, once placed in the appropriate conditions [119,120,121,122,123,124,125,126], and it seems that p53 is no exception to the rule. Indeed, a large body of literature reports that, when subjected to high pressure, high temperature, zinc absence, low pH or RNA molecules, full-length p53, N-terminal, core or C-terminal fragments of p53, can be led to convert into either amorphous aggregates or ThT positive amyloid fibers in vitro [106,113,127,128,129,130,131,132,133,134,135].

The nucleation-dependent mechanism has three characteristics: the existence of a lag time, critical concentration and seeding. The lag time corresponds to the time required for nucleus formation during which the protein appears to be soluble. In contrast with nucleation-dependent polymerization, the growth of a linear polymer does not require nucleation and is characterized by the sequential buildup of intermediates. No lag time is observed, and supersaturated solutions rapidly aggregate. This process can be difficult to distinguish from a nucleation-dependent process with a very short lag time or from seeded growth [136]. The in vitro kinetics of p53 aggregation indeed differs from that of the classical nucleation-growth formation of amyloid fibrils, as the initiation of p53 aggregation happens to be relatively rapid [106,129,137] and as such is reminiscent of that of a linear polymer. The kinetics of p53 aggregation involves the formation of small aggregates that rapidly form amyloid structures that bind ThT and subsequently grow into larger amorphous aggregates. The progress curves fit to a two-step sequential pattern [138,139,140] in which a destabilized mutant p53 may co-aggregate with wild-type p53 and its paralogs p63 and p73. In this model, a mutant preferentially adopts an unfolded structure and would primarily react with another fast-unfolding mutant protein while only occasionally trapping a slow-unfolding wild-type protein. The mutant population rapidly self-aggregates before much of the wild-type p53 protein is depleted. However, as wild-type p53 is incorporated in hetero-tetramers by mutants, the continual production of mutant p53 in a cancer cell would gradually trap more and more wild-type p53 [141], its paralogs p63 and p73 [14,139] and MDM2 [142]. The trapping dynamics could also involve molecular chaperone HSP70 as it is proposed to stabilize mutant p.R175H and increase its aggregation [143]. This may account for the observations of co-aggregates in cell lines and in tumor tissue samples, and possibly causing a dominant-negative effect by directly impairing DNA binding activity [141,144,145].

### 3.3. p53 Aggregation: The Seeding Attempts

As nucleation is rate-limiting at low saturation levels, adding a seed (preformed nucleus) greatly accelerates the polymerization of molecules from solution [120,136,146,147,148,149]. However, in vitro p53 preformed aggregates did not significantly seed the aggregation of bulk proteins and stoichiometric amounts of aggregation-prone mutants induced only small amounts of wild-type p53 to co-aggregate [138,139,140]. p53 seeding experiments are indeed usually based on large amounts of aggregated proteins (10% of the dilution of aggregated proteins) [106] whereas very small amounts of prion or prionoid aggregates (1% to 0.05%) seed aggregation of bulk protein [147,150,151]. In 2013, based on prior demonstration of amyloid aggregation of p53 in vitro, Forget et al. tackled the question of whether or not an in vitro aggregated full-length p53 could be able to propagate its conformation to endogenous wild-type p53 in cells. These p53 aggregates were shown to penetrate cells via a nonspecific macropinocytosis pathway and to induce the co-aggregation of endogenous wild-type p53 in equally amorphous aggregates [152]. However, this report shows a single cell exhibiting p53 aggregates which hardly demonstrates that p53 can induce the aggregation of endogenous p53 proteins. Co-culture experiments were also used by Gosh et al. to demonstrate the cell-to-cell transmission of amyloid aggregates of p53 that were previously induced by the P8 peptide (PILTIITL, corresponding to the residues 250–257 of p53) [113]. The in vitro mechanism of p53 aggregation corresponds more to trapping by cross-reaction and co-aggregation rather than classical seeding and growth (see below; [139]).

### 3.4. p53 Aggregation: The Trapping Evidence

A consequence of prion self-propagation is that PrP^C^ protein overexpression leads to an increased propagation of PrP^Sc^ prion due to the increased availability of its substrate [153,154]. This is also a feature of prionoids. However, the dominant-negative inhibition of mutants and isoforms of p53 has been shown to be dose-dependent in Soas-2 cells [155] as well as in *S. cerevisiæ* [14,156], two models lacking endogenous p53. Conversely, the expression of wild-type p53 has been shown to suppress the growth of tumor cell lines bearing dominant negative p53 mutants [157,158] and to overwhelm the inhibition exerted by mutant p53 on its transcription activity in baker’s yeast [14].

Then, in order to challenge the main ability of prions that is self-propagation, wild-type p53 has been co-expressed in yeast with a mutant p53 (p.R175H or p.R248Q) which expression has been placed under the control of a galactose-inducible promoter in a typical prion-propagation assay [159,160]. When co-expressed with mutant p53, wild-type p53 transcriptional functions were inhibited by the dominant-negative mutant p53. However, as soon as mutant p53 expression was shut off, wild-type p53 fully recovered its transcriptional abilities [14]. These results thus show that mutant p53 did not print its [p53^MUT^] conformation on wild-type p53 in a prion-like manner in *S. cerevisiae*, although recent data also suggest that when fused to EYFP, p53 overexpression leads, in some rare yeast cells, to the formation of aggregates that are transmitted across generations [161]. Altogether, these data show that the dominant-negative effect of mutant p53 is dose-dependent and can be reduced or even neutralized by increasing the wild-type/mutant p53 ratio.

The fact that increasing wild-type p53 expression level can overwhelm mutant p53 dominant-negative effect also led to p53-based gene therapy trials relying on adenoviruses. They aimed at restoring p53 tumor suppressive function in cancer cells by thwarting mutant p53 with a therapeutic wild-type p53 gene. Advexin and Gendicine [162] appeared as the main therapeutic candidates, having been used in numerous trials (including phase III). Chinese FDA approved the use of Gendicine in 2003 for head and neck tumors and in 2005 for naso-pharyngeal cancers [163]. To date, more than 30,000 patients have received Gendicine in association with chemo- or radiotherapy with promising results and relatively few side effects [164].

Other therapeutic strategies aim at reactivating mutant p53 tumor suppressor functions. Quinuclidines PRIMA-1 and its analog APR-246 (phase I and phase II clinical trials) have demonstrated their ability to inhibit cell proliferation and increase apoptosis in cancer cell lines and other models, although it has been reported that they could display non-p53 related activity [165]. The ReACp53 peptide targets the aggregation ability of mutant p53 and restores a partial function of the mutant protein, thereby inducing tumor shrinking both in vitro and in vivo [166,167]. These strategies, among others [168], seem to lead to mutant p53 refolding, allowing for a functional recovery of the protein.

Both the successful therapeutic approaches set up to target mutant p53 and the unsuccessful attempts of propagating [p53^MUT^] conformational phenotype strongly indicate that the p53 protein, be it in a wild-type or mutant state, does not behave as a prion nor as a prionoid.

## 4. Conclusions

Almost 40 years in the making, p53 remains an intense research item, as theories and evidence on its behavior and interactions keep blossoming. From an oncogene, to a tumor suppressor, an amyloid, a prion, p53 has often been studied through the lens of scientific trends [169,170] and once in the air, the idea that p53 behaves in a prion-like manner consistently seeded into the literature.

Although the presented data clearly state that p53 does not behave like a prion or a prionoid, we do not challenge its ability to, potentially, form amyloid fibers in vivo, should concrete evidence be provided. Indeed, human diseases such as Amyloidosis A exhibit amyloid aggregates but no prion or prionoid features [171]. In the case of p53, the formation of amyloid fibers could result from the natural tetramerization between mutant and wild-type p53 serving as a nucleation starting point. Hetero-tetramerization between wild-type p53 and p63/p73 remains unlikely due to the divergence in their tetramerization sequences [20,172]. However, aggregation-prone regions buried in the core domain of p53, which are exposed in several p53 mutants or isoforms, could allow the mutant protein to interact with p63, p73 and MDM2 [14,111,142,173,174,175,176]. These interactions happen preferentially when mutant p53 is able to tetramerize, suggesting that tetramers of mutant p53 may present structural properties allowing them to interact with their paralogs (Figure 2b) [14]. Interaction between p53 and its paralogs is a credible explanation for gain of function properties of p53 mutants evidenced in engineered mice models of Li-Fraumeni Syndrome p53R270H/+ and p53R172H/+ which harbor mutations equivalent to human R273H and R175H, respectively. On the one hand, these mice developed allele-specific tumor spectra distinct from p53+/− mice and on the other hand developed novel tumors when compared to p53−/− mice [177,178].

Given the prevalence of p53 mutations and the frequent accumulation of mutant p53 in cancers, it could be asked whether and how the (co)-aggregation of p53 family members would provide a decisive advantage to malignant cells. Before undergoing loss of heterozygosity, cancerous cells can see a decreased p53 function mainly due to the dominant-negative effect of mutant p53 if the mutant is stabilized. The mechanisms underlying mutant p53 stabilization still need to be elucidated. MDM2 seems indeed able to degrade mutant p53 proteins in vitro and the stabilization of p53 could be related to other factors specifically related to cancerous cell types, the mutation involved and/or loss of heterozygosity itself [105,179]. At later stages, the wild-type allele being often lost, mutant p53 may remain deleterious by trapping p63 and p73 isoforms, thereby maintaining the formation of aggregates. Although induced p53 oligomers have been described as cytotoxic for cells in culture [112] as others types of protein aggregates [85,180], p53 aggregation does not appear to be toxic for cells in vitro nor in vivo, suggesting, once again that there is more to p53 aggregation that meets the eye. Hence, conformational changes, especially amyloid-like, are not necessarily associated with a pathological condition, whereas in the case of prions and prionoids, they definitely are.

In the absence of amyloid characteristics of p53 aggregates, its polymer-like aggregation kinetics, the inability of self-perpetuation of the mutant conformation, and the lack of proven seeding capacities, the qualification of “prion p53” appears largely overstated. The prion p53 theory seems to rather result from an all-too-tempting intellectual jump from the observation of protein aggregates to infectious prion.

## Data Availability

No new data were created or analyzed in this study. Data sharing is not applicable to this article.
